# Sex Differences in Hepatic *De Novo* Lipogenesis with Acute Fructose Feeding

**DOI:** 10.3390/nu10091263

**Published:** 2018-09-07

**Authors:** Wee Suan Low, Thomas Cornfield, Catriona A. Charlton, Jeremy W. Tomlinson, Leanne Hodson

**Affiliations:** 1 Oxford Centre for Diabetes, Endocrinology and Metabolism (OCDEM), University of Oxford, Churchill Hospital, Oxford OX3 7LE, UK; icycool22@hotmail.com (W.S.L.); thomas.cornfield@ocdem.ox.ac.uk (T.C.); mcneil.catriona.a@gmail.com (C.A.C.); jeremy.tomlinson@ocdem.ox.ac.uk (J.W.T.); 2 NIHR Oxford Biomedical Research Centre, University of Oxford, Churchill Hospital, Oxford OX3 7LE, UK

**Keywords:** fructose, liver, lipogenesis, VLDL-TG, sex, human, diet

## Abstract

Dietary free sugars have received much attention over the past few years. Much of the focus has been on the effect of fructose on hepatic de novo lipogenesis (DNL). Therefore the aim of the present study was to investigate the effects of meals high and low in fructose on postprandial hepatic DNL and fatty acid partitioning and dietary fatty acid oxidation. Sixteen healthy adults (eight men, eight women) participated in this randomised cross-over study; study days were separated by a 4-week wash-out period. Hepatic DNL and dietary fatty acid oxidation were assessed using stable-isotope tracer methodology. Consumption of the high fructose meal significantly increased postprandial hepatic DNL to a greater extent than consumption of the low fructose meal and this effect was evident in women but not men. Despite an increase in hepatic DNL, there was no change in dietary fatty acid oxidation. Taken together, our data show that women are more responsive to ingestion of higher amounts of fructose than men and if continued over time this may lead to changes in hepatic fatty acid partitioning and eventually liver fat content.

## 1. Introduction

Dietary sugars have been the focus of much attention and there is now a body of evidence demonstrating that the intake of free sugars or sugar-sweetened beverages is a determinant of body weight [1]. Moreover, a high intake of free sugars may contribute to an increased risk of cardio-metabolic disease, type 2 diabetes and non-alcoholic fatty liver disease (NAFLD), independent of changes in body weight [2]. Of the monosaccharides added to food products, fructose has received the most attention as it is suggested to be the most detrimental to health [3]. However, results from a meta-analysis have suggested fructose has no influence on fasting plasma triacylglycerol (TAG) concentrations unless ≥100 g fructose/day is consumed [4]. As fructose is most commonly consumed in combination with glucose (e.g., high-fructose corn-syrup [5]), it would be reasonable to consider the effects of glucose and fructose together when investigating the effects of free sugars on metabolic health. 

Excess consumption of free sugars has been shown to up-regulate de novo lipogenesis (DNL) and in humans this process primarily occurs in the liver [6]. Dietary fructose has been reported to be more lipogenic than glucose [7]. Work from our group demonstrated that the carbons from labelled (^13^C) fructose, given as a single mixed-test meal, appear in both the fatty-acyl and glycerol moieties of TAG in circulating TAG-rich lipoproteins whilst there is no incorporation of labelled (^13^C) carbons from glucose into these fractions [7]. Other have reported that compared to single bolus of glucose, consumption of an equivalent amount of fructose increases fatty acid markers of postprandial hepatic DNL [8]. Co-ingestion of glucose and fructose have been found to exacerbate hepatic DNL to a greater extent than consumption of the same amount of either glucose or fructose alone [8,9,10]. For example, Parks et al. [9] reported that peak DNL was 7.8% after consumption of glucose whilst after consumption of fructose and glucose in a 1:1 or 3:1 ratio, peak DNL was significantly higher at 15.9%, and 16.9%, respectively. These data suggest that the presence of fructose, with glucose enhances hepatic DNL.

We have previously investigated if there is a sex-specific effect in hepatic DNL between men and women. By studying men and women who were aged, on average 46 years with a mean body mass index (BMI) of 28 kg/m^2^, we found that although fasting hepatic DNL was similar, after consumption of a test meal there was a divergence, with men maintaining a higher contribution of DNL fatty acids to very low-density lipoprotein (VLDL)-TAG than women [11]. Limited work has been undertaken investigating if there are sex-specific effects of fructose intake on hepatic DNL. By studying young (mean age 24 years), lean (mean BMI 22 kg/m^2^) men and women, Tran et al. [12] demonstrated that when given as an acute oral load, the appearance over time, of labelled carbons (^13^C) from fructose into VLDL-TAG palmitate (as a marker of hepatic DNL), was significantly higher compared to women. Taken together, these findings suggest that men, irrespective of age and BMI appear to have higher hepatic postprandial DNL and are more responsive to fructose intakes than age and BMI matched women.

In-line with men being reported to have higher postprandial hepatic DNL than women [11,12]; white men have a higher prevalence of NAFLD compared to white women [13,14]. It is often suggested that enhanced hepatic DNL plays a key role in the development of NAFLD as it shifts cellular metabolism away from fatty acid oxidation towards esterification [15]. This shift occurs due to an elevation in malonyl-CoA, an intermediate in the DNL pathway, inhibiting carnitine palmitoyltransferase I (CPT1) which is required for the transport of fatty acyl-CoAs from the cytoplasm to the mitochondria for oxidation [16]. We [11,17] and others [18] have previously reported inverse associations between hepatic DNL and a proxy marker of hepatic fatty acid oxidation, plasma 3-hydroxybutyrate (3-OHB). From the limited data available where fructose has been fed and markers of fatty acid oxidation measured, it was found that postprandial plasma 3-OHB concentrations were significantly lower after consumption of a meal containing fructose compared to glucose [7] or after a 6-day isocaloric diet that was supplemented with fructose [19]. Moreover, net fat oxidation has been reported to decrease and net carbohydrate oxidation increase to a greater extent after fructose than after glucose consumption [7] and after 6 days of a fructose-enriched diet [19]. It remains unclear how much fructose is required to be consumed before alterations in hepatic DNL and fatty acid oxidation are evident.

Therefore, the aim of the present study was to investigate the effect of a single meal that was high in fructose compared to a meal low in fructose on postprandial hepatic DNL, fatty acid partitioning and dietary fatty acid oxidation in healthy men and women.

## 2. Materials and Methods

### 2.1. Subjects

Sixteen healthy adults (eight men, eight women) were recruited from the Oxford BioBank [20] for this randomized cross-over study, with study days being separated by a 4-week wash-out period. All volunteers were considered non-diabetic and free from any known disease, were not taking medication known to affect lipid or glucose metabolism, did not smoke, and did not consume alcohol above recommended limits [21]. The study was approved by Portsmouth Clinical Research Ethics Committee (Portsmouth REC 12/SC/0267); all subjects gave written informed consent. The study was registered with clinical trials.org (NCT02478541).

### 2.2. Liver Fat and Body Composition

Intra-hepatic fat was measured after an overnight fast, and within 2 weeks of the metabolic study day, using proton magnetic resonance spectroscopy [22] and whole body composition and fat quantification, distribution were measured using dual-energy X-ray absorptiometry (DEXA) [23].

### 2.3. Metabolic Study Days

Prior to the study days, subjects were asked to avoid foods naturally enriched in ^13^C (e.g., corn based products such as cornflakes) and to refrain from strenuous exercise and alcohol. Participants arrived at the clinical research unit after an overnight fast and after consuming deuterated water (^2^H_2_O) (3 g/kg body water) the evening prior to the study day and then consumed enriched water (2.5 g per 500 mL water) until the end of the study day, in order to achieve and maintain a plasma water enrichment of 0.3 % for the measurement of fasting and postprandial hepatic DNL. A cannula was placed in an antecubital vein and baseline (time, *t* = 0 min) blood and breath samples were taken.

On the study day, participants were fed a meal consisting of two drinks; a chocolate drink and a lemon-flavoured sugar drink. The chocolate drink contained 50 g fat (30 g of palm oil and 20 g of olive oil) and 200 mg (U^13^C) palmitic acid to trace the fate of the dietary fatty acids. The composition of the chocolate drink was the same for both study days and had a ratio of 40:50:10 of saturated, monounsaturated and polyunsaturated fatty acids, which mimicked the physiological ratio of fatty acids in human blood [24]. The lemon-flavoured sugar drink contained 100 g monosaccharides on both study days but varied in composition: (1) a low fructose drink consisting of 20 g fructose and 80 g glucose or (2) a high fructose drink containing 60 g fructose and 40 g glucose; subjects consumed these in random order. As fructose and glucose are rarely consumed in isolation and previous studies had given fructose and glucose either in isolation or in a ratio of 1:1 [8,9] or 3:1 [9], we decided to give a lower amount for the low fructose meal (ratio 1:4 of fructose: glucose) and then in proportion to that would more closely mimic high fructose corn syrup (1.5:1 fructose: glucose).

After consumption of the test meal, repeated blood and breath samples were taken over the course of the study period. Indirect calorimetry was performed 120 min after the meal consumption using a Gem calorimeter to determine whole body CO_2_ production (GEMNutrition Ltd., Daresbury, Cheshire, UK).

### 2.4. Biochemical Analysis

Whole blood was collected into heparinized syringes (Sarstedt Ltd., Leicester, UK) and plasma was separated by centrifugation. Plasma metabolites and insulin were analyzed as previously described [25].

Separations of Svedberg flotation rate (*S_f_*) > 400 (“chylomicrons”) and TAG-rich lipoprotein (TRL) fraction (*S_f_* 20–400) were made by sequential flotation using density gradient ultracentrifugation as previously described [26]. The *S_f_* 20–400 fraction was further separated by immunoaffinity chromatography to obtain a fraction completely devoid of apoB48 and will hereafter be called VLDL [26].

Blood samples were taken at 0, 30, 60, 90, 120, 180, 240, 300 and 360 min after the consumption of the test meal for the measurement of plasma glucose, fructose, insulin, TAG, non-esterified fatty acids (NEFA), 3-OHB and at 0, 120, 180, 240, 300 and 360 min for the analysis of chylomicron-TAG, TRL-TAG and VLDL-TAG. Breath samples were collected at 0, 30, 60, 90, 120, 180, 240, 300 and 360 min into EXETAINER^®^ tubes (Labco Ltd., High Wycombe, Bucks, UK) for measurement of ^13^CO_2_ enrichment.

### 2.5. Fatty Acid Analysis and Isotopic Enrichment

To determine specific fatty acid composition and isotopic enrichment, total lipids were extracted from plasma and lipoproteins. Fatty acid methyl esters (FAMEs) were prepared from NEFA and TAG fractions as previously described [27]. Fatty acid compositions (µmol/100 µmol total fatty acids) in these fractions were determined by gas chromatography (GC) [28], and palmitate concentrations were calculated by multiplying the proportion of palmitate by the corresponding plasma concentrations of NEFA, TAG or lipoprotein-TAG determined enzymatically.

^13^C/^12^C ratios in (U^13^C)palmitate in plasma NEFA, TAG, *S_f_* > 400 (chylomicron-TG), *S_f_* 20–400-TAG (TRL) and VLDL-TAG FAMEs derivatives and the ^13^C/^12^C ratio in breath samples were measured as previously described [7]. Tracer to tracee ratios (TTRs) at baseline (time 0) was subtracted from each sample TTR to account for natural abundance. The TTRs for (U^13^C) palmitate were multiplied by the corresponding palmitate-NEFA or palmitate-TAG concentrations to give plasma and lipoprotein tracer concentrations. The relative rate of whole-body meal-derived fatty acid oxidation was calculated by multiplying the CO_2_ production (VCO_2_, mmol/min) by the TTR of ^13^CO_2_/^12^CO_2_ [7]. When comparing differences between men and women, we corrected the data for lean mass (determined by DEXA).

Fasting and postprandial hepatic DNL was assessed and this was based on the incorporation of deuterium from ^2^H_2_O in plasma water (Finnigan GasBench-II, ThermoFisher Scientific, UK) into VLDL-TAG palmitate using GC-Mass Spectrometry (GC-MS) with monitoring ions with mass-to-charge ratios (m/z) of 270 (M + 0) and 271 (M + 1) [29]. This represents the synthesis of fatty acids from precursors such as sugars and amino acids [30].

### 2.6. Calculations and Statistical Analysis

We have previously observed a difference in postprandial hepatic DNL between men and women [11] and this study provided the opportunity to investigate if there is a sex-specific response between the low and high fructose meals. Based on our previous observation of a difference in hepatic DNL fatty acids in VLDL-TAG of 4.2 ± 2.9% between men and women at the end of the postprandial period, the number of men and women required to detect the same difference with a power of 0.80 at α of 0.05 was *n* = 8 per group.

Homeostatic model assessment of insulin resistance (HOMA-IR) was calculated [31]. The relative and absolute contribution of meal fatty acids to VLDL-TAG was calculated at the end of the study (Time 360 min) [32].

Data were analysed using SPSS for Windows v22 (SPSS, IBM, Chertsey, UK). Statistical significance was set at *P* < 0.05. All data are presented as mean ± standard error of the mean (SEM) unless stated. Data sets were tested for normality according to the Shapiro-Wilk test. Areas under the curve (AUCs) were calculated by the trapezoid method. AUCs have been divided by the relevant time period to give time-averaged values. Comparisons in anthropometric and fasting parameters between the test meals were made using a Students paired *t*-test and comparisons between meal and between sexes made using an independent *t*-test. Repeated measures analysis of variance (ANOVA), with time and test meal as factors, was used to investigate the change between the meals over time and then repeated measures ANOVA, with time and sex as factors, were used to investigate the change between sexes for the same test meal. Comparisons were made after adjustment for baseline values. Correlations were performed using Spearman Rank Correlation.

## 3. Results

### 3.1. Participant Characteristics

Sixteen apparently healthy participants, who were free from known disease and not taking any medication known to alter glucose or lipid metabolism, were recruited into the study. Men and women were similar in age, BMI, waist circumference, total and visceral fat mass and liver fat (Table 1). Women had significantly (*P* < 0.01) more gynoid fat, a significantly (*P* < 0.05) lower android to gynoid fat ratio and a significantly (*P* < 0.001) lower amount total lean mass than men (Table 1).

**Figure 1:**
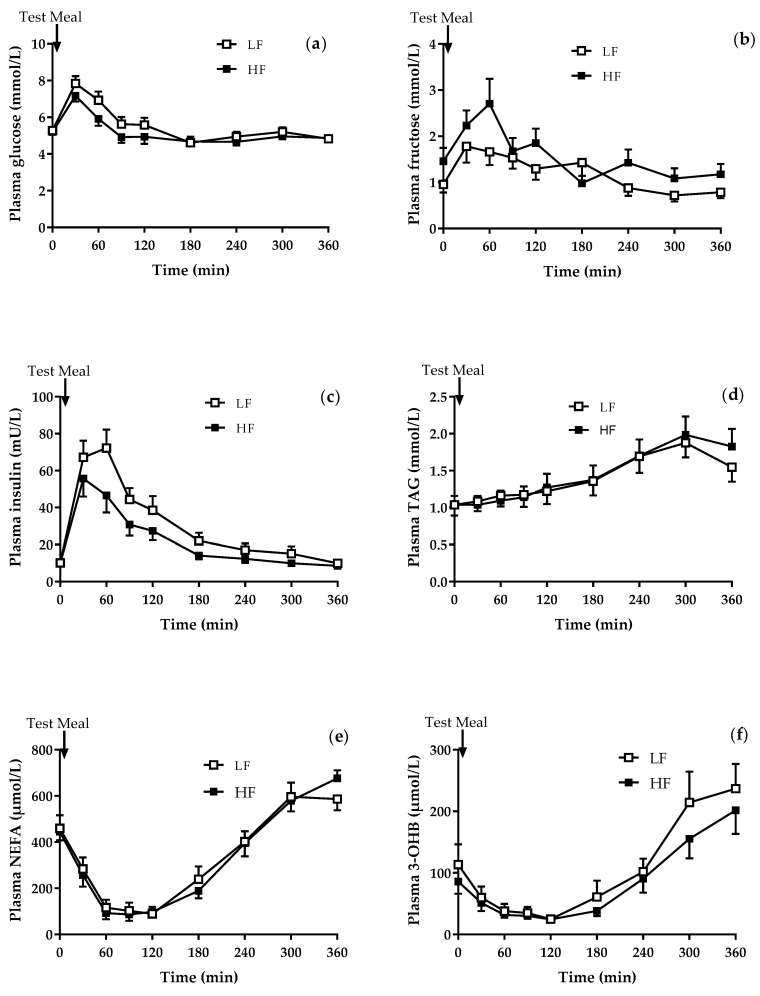
The postprandial response after the consumption of the low fructose (LF) and high fructose (HF) meals of (**a**) plasma glucose (*P* < 0.001 time, *P* < 0.01 meal); (**b**) plasma (*P* < 0.001 time, *P* < 0.05 meal); (**c**) plasma insulin (*P* < 0.001 time, *P* < 0.01 meal), (**d**) plasma triacylglycerol (TAG) (*P* < 0.001 time); (**e**) plasma non-esterified fatty acids (NEFA) (*P* < 0.001 time); and (**f**) plasma 3-hydroxybutyrate (3-OHB) (*P* < 0.001 time). Data presented as mean ± standard error of the mean (SEM).

Each participant had 2 study days, approximately 4 weeks apart and consumed the study meal (a chocolate drink and a lemon-flavoured sugar drink either low or high in fructose) in random order. Between the 2 study days, there was no difference in the body weight, nor were there any differences in fasting plasma concentrations of glucose, fructose, insulin, cholesterol, NEFA, TAG and 3-OHB (Table 2). There was a small, but significant (*P* < 0.05) difference in plasma HDL-cholesterol concentrations which were higher on the high fructose compared to low fructose study day (Table 2). There was a significantly (*P* < 0.01) lower postprandial response in plasma glucose and insulin after the high compared to the low fructose meal, whilst there was a significantly (*P* < 0.05) higher response in plasma fructose after the high compared to low fructose meal (Figure 1a–c). There was no difference in the postprandial response of plasma TAG, NEFA, and 3-OHB betweent thelow and high fructose meals (Figure 1d–f). The postprandial response of chylomicron-TAG, TRL-TAG and VLDL-TAG were also similar after consumption of the low and high fructose meals (Table 2).

There was no difference in BMI (kg/m^2^) between men and women on either of the study days; body weight also remained unchanged (Table 2). There was no difference for the majority of fasting plasma metabolites between the low and high fructose study days between men and women; however, fasting plasma insulin concentrations were significantly (*P* < 0.05) lower in women compared to men on the low fructose study day, which translated into a significantly (*P* < 0.05) lower HOMA-IR (Table 2). After consumption of the low fructose meal, the only metabolic parameter to differ between sexes was the excursion of chylomicron-TAG, which was significantly (*P* < 0.05) lower in women compared to men (Table 2).

There were no notable differences within either sex in fasting plasma biochemical parameters between the low and high fructose study days (Table 2). Consumption of the high fructose meal elicited similar postprandial responses in the respective biochemical variables in both men and women. However in women, consumption of the high fructose meal resulted in significantly (*P* < 0.05) lower plasma glucose and insulin excursions compared to the low fructose meal; this was not evident in men. There were no within-sex differences in the postprandial response of plasma fructose, TAG and NEFA. There was no signficiant difference in the postprandial response of plamsa 3-OHB between the meals and none within sex differences; however, there was a significant (*P* < 0.05) time × sex interaction after the low fructose meal, with women having a notably higher rebound (after nadir) than men (data not shown).

### 3.2. Dietary Fatty Acid Partitioning and Substrate Utilization

We included (U^13^C)palmitate in the chocolate drink, that was consumed with the low and high fructose drinks, to trace the fate of dietary fatty acids. We found that there was a tendency (*P* ≤ 0.07) for a lower appearance of ^13^C (from dietary fat) into the plasma NEFA pool (Table 3). If hepatic DNL differed in response to the different fructose meals, this may alter cellular metabolism with an upregulation of DNL shifting cellular metabolism toward esterification (for storage or secretion in VLDL-TAG) and away from oxidation pathways. Therefore, we calculated the relative contribution of dietary fatty acids to VLDL-TAG and found there was a significantly (*P* < 0.05) lower contribution after consumption of the high compared to low fructose meal (Table 3).

There was a lower appearance of ^13^C from dietary fat in the plasma NEFA pool when women consumed the high fructose meal only, whilst consumption of the high fructose meal resulted in a lower relative contribution of dietary fatty acids to VLDL-TAG in men, but not women (Table 3); there was no difference in the absolute concentration of dietary fatty acids to VLDL-TAG in either sex (data not shown). Consumption of the low fructose meal resulted in a significantly (*P* < 0.05) lower appearance of ^13^C-pamitate in chylomicron-TAG in women compared to men whilst the incorporation of ^13^C into expired CO_2_ was significantly lower after consumption of the high fructose meal in women compared to men; however, after correcting for differences in lean mass between the sexes, this difference disappeared (Table 3). We assessed the participants respiratory exchange ratio (RER) 120 min after consumption of the meals and found a trend (*P* = 0.07) for a higher RER after consumption of the high compared to low fructose meal (0.88 ± 0.01 vs. 0.82 ± 0.02, respectively). There were no differences in RER between or within sexes after consumption of the meals.

**Figure 2:**
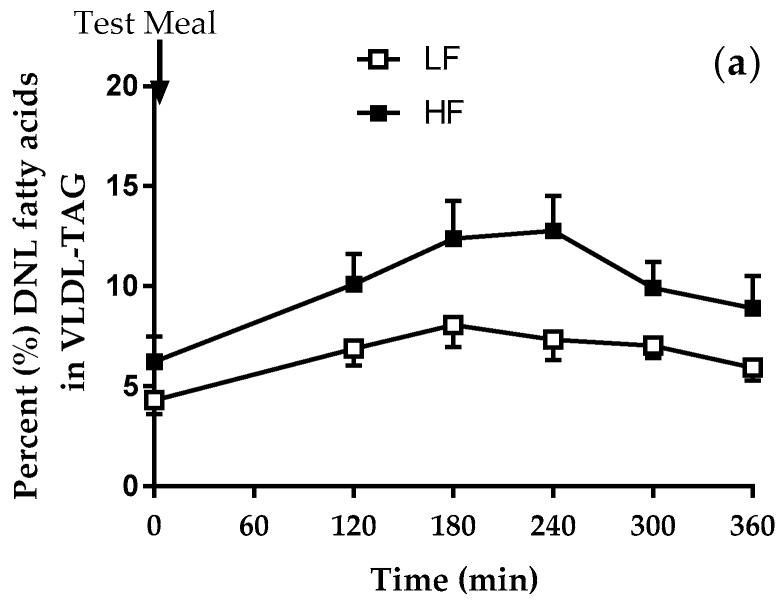

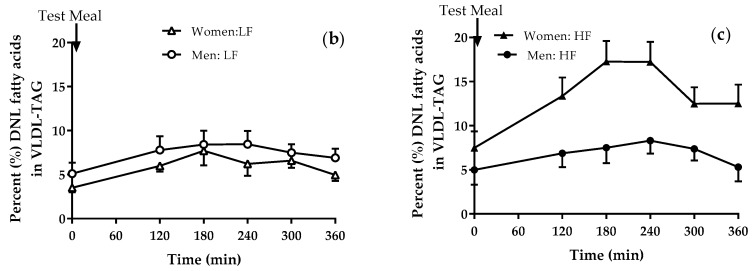
The contribution (%) of DNL fatty acids to VLDL-TAG in (**a**) the whole group after the consumption of the low fructose (LF) and high fructose (HF) meals (*P* < 0.001 time, *P* = 0.05 meal, time × meal *P* = 0.06); (**b**) women and men after consumption of the low fructose (LF) meal (*P* < 0.001 time); and (**c**) women and men after consumption of the high fructose (HF) meal (*P* < 0.001 time, *P* < 0.05 sex, *P* < 0.01 time × sex interaction). Data presented as mean ± SEM.

The relative contribution of DNL fatty acids in VLDL-TAG was assessed in the fasting and postprandial state on each of the respective study days. Although there was a significant (*P* < 0.001) increase in the contribution of DNL fatty acids in VLDL-TAG after consumption of both meals, the contribution was significantly (*P* < 0.05) higher after consumption of the high compared to low fructose meal (Figure 2a). After consumption of the low fructose meal, the relative contribution of DNL fatty acids in VLDL-TAG increased (*P* < 0.001) to a similar extent in both sexes (Figure 2b). In contrast, after consumption of the high fructose meal there was a notable difference in the contribution of DNL fatty acids in VLDL-TAG, with women having a more evident response than men (time *P* < 0.001, sex *P* < 0.001, time × sex interaction *P* < 0.05) (Figure 2c). After men consumed the low and high fructose meals there was no difference in the contribution of DNL fatty acids in VLDL-TAG whilst for women the contribution was significantly greater after consumption of the high compared to low fructose meal (time *P* < 0.001, meal *P* < 0.001, time × meal interaction *P* < 0.01).

### 3.3. Associations Between Hepatic DNL and Markers of Postprandial Fatty Acid Oxidation

**Figure 3:**
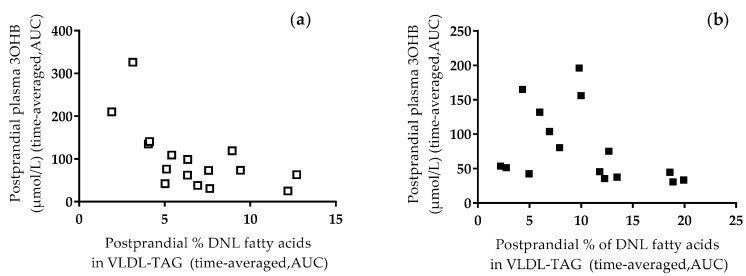
The association between the postprandial contribution (%) of DNL fatty acids in VLDL-TAG and the postprandial plasma concentrations of 3-hydroxybutyrate (3-OHB) after (**a**) the low fructose meal, and (**b**) the high fructose meal.

We assessed the association between the postprandial contribution (%) of DNL fatty acids in VLDL-TAG and postprandial concentrations of plasma 3-OHB (as a surrogate marker of hepatic fatty acid oxidation) and found inverse associations after the low fructose meal (*r_s_* = −0.66, *P* < 0.01 Figure 3a) and the high fructose meal (*r_s_* = −0.57, *P* < 0.05, Figure 3b). The association between the postprandial contribution (%) of DNL fatty acids in VLDL-TAG and postprandial concentrations of plasma 3-OHB after the low fructose meal was significant for men (*r_s_* =−0.71, *P* < 0.05) and not women (*r_s_* = −0.62, *P* = NS). In contrast, the association between these variables was only significant in women after consumption of the high fructose meal (*r_s_* = −0.88, *P* < 0.01) but not in men (*r_s_* = −0.45, *P* = NS).

## 4. Discussion

Dietary free sugars are typically derived from the two most commonly consumed monosaccharides, glucose and fructose. Despite having the same empirical formula, glucose and fructose differ in their absorption, insulin response and effect on hepatic DNL [3,33,34]. Although the effects of dietary fructose on hepatic DNL are well documented [7,8,9,10,12,35], the effect of fructose consumption on dietary fatty acid oxidation remains unclear. Therefore, we assessed the effects of single meals that were low and high in fructose on postprandial hepatic DNL, fatty acid partitioning and dietary fatty acid oxidation. Overall, we found consumption of the high fructose meal resulted in a greater proportion of DNL fatty acids in VLDL-TAG than consumption of the low fructose meal and this effect was evident in women but not men; there was no effect on dietary fatty acid oxidation with either meal. 

The reported average daily intake of free sugars by UK adults is approx. 50 g/day [36] and fructose contributes up to 60% [37]. Therefore, we designed the high fructose meal to recapitulate the contribution of fructose whereby it contained 60 g fructose and 40 g glucose whilst the low fructose meal contained 20 g fructose and 80 g glucose. In-line with previous findings [7,8,9,35], we found the high fructose meal to have a greater effect on hepatic DNL than the low fructose meal. Within the liver, insulin activates the transcription factor sterol regulatory element-binding protein 1c (SREBP-1c) which enhances the transcription of genes required for fatty acid and TAG synthesis [38,39]. Despite the low fructose meal resulting in significantly higher postprandial plasma glucose and insulin excursions when compared to the high fructose meal, hepatic DNL was lower. A plausible reason why hepatic DNL was significantly increased after the high fructose meal is based on the suggestion that fructose may act indirectly to stimulate SREBP-1c and carbohydrate-responsive element-binding protein (ChREBP) via PPAR-γ coactivator 1β which is a transcriptional coactivator for SREBP-1c [40]. Additionally, fructose-1,6-bisphosphate, an intermediate in the fructose metabolic pathway, can combine with glyceraldehyde to produce xylulose-5-phosphate [41], which is a strong activator of protein phosphatase 2A [42] and can activate ChREBP [43]. Thus, it is likely that the combined effect of insulin and fructose activating SREBP-1c directly and indirectly led to the notably higher induction of hepatic DNL after the high compared to low fructose meal.

We have previously found that men have higher postprandial hepatic DNL when compared to age and BMI matched women [11]. This observation is in line with findings from animal work which have suggested a role for oestrogen in attenuating hepatic DNL, with acetyl-CoA carboxylase activity being reduced with oestrogen treatment in female rats [44]; oophorectomy has been reported to increase the expression of SREBP-1c and liver TAG content [45]. However, our findings here demonstrate that when women are given a high fructose meal, the contribution of DNL fatty acids in VLDL-TAG is notably higher than men. This is in contrast to the results of Tran et al. [12] who reported that after an acute oral fructose load, markers of DNL were significantly higher in men compared to women. Why the women in the present study had a dramatically different response compared to men is not clear and intriguing. In support of our findings, work in a rodent model feeding a high-fat, high-fructose diet (HFFD) to male and female Sprague-Dawley rats for 12 weeks found that the hepatic protein level of acetyl-CoA carboxylase and fatty acid synthase was significantly upregulated in female rats on the HFFD compared to the control diet whilst there was no change in male rats between the HFFD and control diets [46]. As there are only a limited number of studies that have been undertaken to date, further work is required to understand the conflicting nature of the sex-specific response in hepatic DNL to fructose ingestion.

The measurement of hepatic DNL, in vivo in humans is reliant on DNL fatty acids being incorporated into VLDL-TAG that is then secreted into systemic circulation. Therefore, it is plausible that men had an upregulation of hepatic DNL after consumption of the high fructose meal to a similar or greater extent than women but rather than being secreted in VLDL-TAG it was partitioned toward a cytosolic TAG storage pool and remained in the liver. Previous work undertaken in eight healthy men has reported that peak DNL occurred after consumption of a second meal [47], suggesting there is a time delay for the sensitization of the liver to upregulate lipogenic enzymes; studies of a similar nature have not been undertaken in women. 

In contrast to previous work from the authors [7], no difference was found in postprandial plasma 3-OHB concentrations or pattern of response after consumption of the high fructose compared to low fructose meal. However, we did find a difference in the pattern of response between men and women after consumption of the low fructose meal, whereby women had a notably greater and more accelerated rebound in plasma 3-OHB concentrations than men. This is in line with our previous observation of women having a higher postprandial plasma 3-OHB concentration (after nadir) than men [11].

By incorporating ^13^C into the meal we were able to trace the fate of dietary fatty acids and determine rates of expiration of ^13^CO_2_ in breath. We found no between-meal or within or between sex differences in the rate of expiration of ^13^CO_2_ (as a marker for whole-body dietary fatty acid oxidation), despite an increase in hepatic DNL after the consumption of a high fructose meal. We did however, observe a trend for a higher postprandial RER after consumption of the high fructose compared to the low fructose meal, implying that carbohydrate, rather than fat, was being utilised as fuel. This is in agreement with the observation of Chong et al. [7] who suggested the higher postprandial RER after fructose rather than glucose consumption indicated fatty acids were being channelled toward esterification, rather than oxidation [7]. Although we did not find a sex-specific effect for RER after the respective meals, Tran et al. [12] have previously reported that following fructose ingestion, net fat oxidation was significantly suppressed whereas net carbohydrate oxidation was significantly increased in men whereas in women neither net fat nor carbohydrate oxidation was changed. We did find inverse associations between the postprandial contribution of DNL fatty acids in VLDL-TAG and the postprandial concentrations of plasma 3-OHB suggesting that when hepatic DNL was increased then ketogenesis was attenuated. This association was only significant in men after consuming the low but not high fructose meal and only significant in women after consuming the high but not low fructose meal. The divergence in these results suggests sex-specific differences in the regulation of these pathways.

Although there was no difference in postprandial systemic NEFA concentrations between meals or between or within sexes, there was a significant decrease in the appearance of ^13^C from dietary fat, into the systemic NEFA pool after consumption of the high fructose meal. The appearance of ^13^C in the plasma NEFA pool is consistent dietary fatty acid spillover [48] and adipose tissue lipoprotein lipase (LPL) plays a key role in the disposal of dietary fatty acids [49]; in the postprandial state, LPL is upregulated as part of an insulin-mediated response [50]. Thus, the lower appearance of ^13^C from dietary fat in the systemic NEFA pool after the high fructose meal may in part be explained by the notably lower insulin concentrations.

Postprandial plasma fructose concentrations were substantially lower than plasma glucose concentrations, despite a greater amount of fructose (in the high fructose meal) being consumed; this has previously been observed [7,12]. Although it is often suggested that the liver is the main organ for fructose metabolism, it has recently been demonstrated in an animal model that the small intestine metabolizes dietary fructose into glucose and organic acids [51]. Evidence for this conversion in humans is required but it is tempting to speculate that when fructose is consumed, it is converted to glucose and other organic acids within the small intestine and therefore does not have such a profound excursion in systemic circulation, leading to a lower metabolic effect on the liver. However, when the intestinal capacity for fructose conversion is saturated, then it is likely the liver will be exposed to a greater concentration of fructose [51]. 

Our study is not without limitations. We did not standardize participants’ diets before the first study day or over the 4-week washout period. Although participants were advised to maintain regular dietary and lifestyle habits, it is possible that they may have altered what they typically did; however, no notable differences between study days were evident. We only measured liver fat once over the course of the study and it is plausible that small changes in liver fat occurred between study days that may have influenced hepatic DNL and fatty acid partitioning. We investigated the acute, rather than the chronic, effect of consuming a single meal which was high or low in fructose in combination with dietary fat on hepatic DNL and fatty acid partitioning. It is likely that if participants continued consuming high fructose meals that changes in plasma variables, including TAG and glucose, would have become evident. Previous work by Couchepin et al. [19] found that consumption of a 6-day isocaloric diet supplemented with 3.5 g fructose/kg lean mass resulted in significant increases in plasma glucose and TAG concentrations, in conjunction with a significant decrease in plasma 3-OHB when compared to a control diet and these changes were more evident in men than women. Finally, the participants in this study were Caucasian men and women, aged between 38 to 50 years, who were in good health and had a BMI (on average) around 29 kg/m^2^ ; therefore, it remains unclear if the results observed here can be translated to other populations.

## 5. Conclusions

The effect of free sugars, particularly fructose, has received a lot of attention over the past few years not least for the effect it has on hepatic DNL. There is clear evidence that fructose is more lipogenic than glucose and this is exacerbated when fructose and glucose are co-ingested and we found, it appears to have a sex-specific response. Based on the data presented, women are more responsive to ingestion of higher amounts of fructose with glucose than men, which is in contrast to the findings of others [12,19]. Although it remains unclear if there is a sex-specific response in hepatic DNL, it is likely that if a fructose-enriched diet is consumed chronically, this may lead to changes in hepatic fatty acid partitioning and an accumulation of liver fat content. 

## Figures and Tables

**Table 1:** Participant characteristics.

Age (y)	Men (*n* = 8)	Women (*n* = 8)
	42.8 ± 1.8	46.6 ± 0.9
Weight (kg)	93.0 ± 3.3	81.0 ± 3.3 *
Height (cm)	178 ± 2	169 ± 2 **
BMI (kg/m^2^)	29.2 ± 0.9	28.5 ± 1.2
Waist circumference (cm)	101 ± 4	94 ± 4
Hip circumference (cm)	106 ± 1	110 ± 2
Waist to hip ratio	0.95 ± 0.03	0.86 ± 0.03 *
Total fat mass (kg)	27.7 ± 2.6	33.7 ± 3.1
Total lean mass (kg)	61.6 ± 1.3	44.7 ± 1.7 ***
Android fat (kg)	2.8 ± 0.4	2.8 ± 0.5
Gynoid fat (kg)	4.0 ± 0.3	5.7 ± 0.4 **
Android/Gynoid fat ratio	0.7 ± 0.1	0.5 ± 0.1 *
Visceral fat (kg)	1.3 ± 0.3	0.9 ± 0.3
Liver fat (%)	3.1 ± 1.1	3.3 ± 1.6

Data presented as mean ± standard error of the mean (SEM). Abbreviations: Body Mass Index, BMI. * *P* < 0.05, ** *P* < 0.01, *** *P* < 0.001 men vs. women.

**Table 2:** Participant characteristics and biochemical variables on the low-fructose and high-fructose meal study days.

	Low Fructose Meal			High Fructose Meal		
	All (*n* = 16)	Men (*n* = 8)	Women (*n* = 8)	All (*n* = 16)	Men (*n* = 8)	Women (*n* = 8)
Weight (kg)	87.7 ± 2.8	93.5 ± 3.2	81.2 ± 3.3 ^†^	87.3 ± 2.7	93.6 ± 3.3	81.9 ± 3.5 ^†^
BMI (kg/m^2^)	29.0 ± 0.7	29.4 ± 0.8	28.6 ± 1.1	29.2 ± 0.7	29.4 ± 0.9	29.0 ± 1.1
HOMA-IR	2.4 ± 0.3	3.0 ± 0.3	1.8 ± 0.3 *	2.3 ± 0.2	2.7 ± 0.4	1.9 ± 0.2
***Fasting Plasma Biochemical Parameters***						
Glucose (mmol/L)	5.3 ± 0.2	5.5 ± 0.3	5.0± 0.2	5.2 ± 0.1	5.3 ± 0.1	5.1 ± 0.3
Fructose (mmol/L)	0.2 ± 0.0	0.2 ± 0.1	0.2 ± 0.1	0.3 ± 0.1	0.2 ± 0.1	0.3 ± 0.1
Insulin (mU/L)	10.1 ± 0.9	12.0 ± 1.0	8.2 ± 1.1 ^†^	9.9 ± 1.0	11.6 ± 1.5	8.5 ± 1.0
Total Chol (mmol/L)	4.7 ± 0.2	4.6 ± 0.2	4.8 ± 0.4	4.9 ± 0.2	4.8 ± 0.3	5.1 ± 0.4
HDL-Chol (mmol/L)	1.3 ± 0.1	1.2 ± 0.1	1.5 ± 0.3	1.4 ± 0.1 *	1.3 ± 0.1	1.6 ± 0.2
Non-HDL-Chol (mmol/L)	3.4 ± 0.2	3.4 ± 0.3	3.3 ± 0.2	3.5 ± 0.2	3.5 ± 0.3	3.5 ± 0.2
NEFA (µmol/L)	459 ± 57	383 ± 56	536 ± 95	445 ± 38	416 ± 32	474 ± 69
TAG (mmol/L)	1.0 ± 0.1	1.2 ± 0.3	0.9 ± 0.1	1.0 ± 0.1	1.1 ± 0.2	1.0 ± 0.1
3-OHB (µmol/L)	114 ± 33	115 ± 64	112 ± 24	86 ± 20	98 ± 33	74 ± 23
***Time-averaged postprandial plasma concentrations***						
Chylomicron-TAG (µmol/L)	229 ± 33	321 ± 41	138 ± 24 ^††^	256 ± 61	347 ± 110	164 ± 37
TRL-TAG (µmol/L)	711 ± 102	860 ± 178	563 ± 82	784 ± 117	890 ± 202	677 ± 120
VLDL-TAG (µmol/L)	345 ± 43	383 ± 77	308 ± 40	343 ± 43	347 ± 68	338 ± 57

Data presented as mean ± SEM. Abbreviations: BMI, Body Mass Index; HOMA-IR, homeostatic model assessment of insulin resistance; Chol, Cholesterol; HDL, high density lipoproteins; NEFA, non-esterified fatty acids, TAG, triacylglycerol; TRL, triacylglycerol-rich lipoproteins; VLDL, very low density lipoproteins; 3-OHB, 3-hydroxybutyrate. * *P* < 0.05, low vs. high fructose; **^†^** *P* < 0.05, **^††^** *P* < 0.01 men vs. women.

**Table 3:** The time-averaged postprandial incorporation of ^13^C (from dietary fat) into plasma and breath and the contribution of dietary fat to VLDL-TAG after consumption of a low- and high-fructose meal.

	Low Fructose Meal			High Fructose Meal		
	All (*n* = 16)	Men (*n* = 8)	Women (*n* = 8)	All (*n* = 16)	Men (*n* = 8)	Women (*n* = 8)
(^13^C) palmitate in chylomicron-TAG (µmol/L)	0.87 ± 0.12	1.1 ± 0.1	0.6 ± 0.2 ^†^	0.88 ± 0.22	1.2 ± 0.4	0.6 ± 0.1
(^13^C) palmitate in plasma NEFA (µmol/L)	0.42 ± 0.06	0.43 ± 0.09	0.41 ± 0.08	0.32 ± 0.04	0.35 ± 0.04	0.30 ± 0.07 ^‡^
(^13^C) palmitate in VLDL-TAG (µmol/L)	0.09 ± 0.01	0.10 ± 0.02	0.07 ± 0.02	0.07 ± 0.01	0.09 ± 0.02	0.06 ± 0.01
(^13^C) palmitate in plasma TAG (µmol/L)	1.62 ± 0.21	2.1 ± 0.3	1.1 ± 0.2	1.30 ± 0.21	1.6 ± 0.4	1.0 ± 0.2
^13^CO_2_ (µmol/min)	1.6 ± 0.2	1.8 ± 0.3	1.3 ± 0.2	1.5 ± 0.2	1.8 ± 0.3	1.1 ± 0.1 ^†^
^13^CO_2_ (µmol/min/kg lean mass)	0.03 ± 0.004	0.03 ± 0.001	0.03 ± 0.001	0.03 ± 0.003	0.03 ± 0.004	0.03 ± 0.003
Relative (%) contribution of dietary fat to VLDL-TAG	23 ± 2	26 ± 3	20 ± 3	19 ± 2 *	21 ± 3 ^‡^	17 ± 2

Data presented as mean ± SEM. Abbreviations: NEFA, non-esterified fatty acids, TAG, triacylglycerol; VLDL, very low density lipoproteins; 3-OHB, 3-hydroxybutyrate. * *P* < 0.05 low vs high fructose; **^†^** *P* < 0.05 men vs women; **^‡^** *P* < 0.05 within sex low vs high fructose.
